# Ionic Homeostasis Failure in Major Depressive Disorder: Ion Channel Mechanisms, Excitation–Inhibition Imbalance, and Precision Therapeutics

**DOI:** 10.3390/ijms27136084

**Published:** 2026-07-07

**Authors:** Yohan Seo

**Affiliations:** Department of Physiology, Dongguk University College of Medicine, Gyeongju 38066, Republic of Korea; ddukdae12@dongguk.ac.kr

**Keywords:** major depressive disorder, ionic homeostasis, ion channels, excitation–inhibition balance, neuroinflammation, glial channels, ketamine, precision psychiatry

## Abstract

Major depressive disorder (MDD) remains a leading cause of disability; however, monoaminergic models do not fully explain delayed treatment onset, incomplete remission, or rapid responses to glutamatergic interventions. In this study, we proposed a system-level ionic homeostasis framework for MDD. In this model, genetic susceptibility, chronic stress, metabolic burden, and neuroinflammation converge in neuronal and glial ion-channel systems, disrupting calcium, potassium, chloride, and purinergic homeostasis. These disturbances alter intrinsic excitability, synaptic integration, inhibitory tone, glial buffering, and neuron–glia signaling, thereby promoting excitation–inhibition imbalance, impaired plasticity, and corticolimbic network instability. We reviewed the evidence implicating the CACNA1C/Cav1.2, TREK-1, KCNQ, NKCC1/KCC2, HCN, transient receptor potential/acid-sensing ion channels, and glial mediators, including P2X7R, Kir4.1, and AQP4. We also discuss how ketamine-related mechanisms, chloride-restoring strategies, anti-inflammatory ion channel targeting, neuromodulation, EEG biomarkers, and AI/multiomics approaches support mechanism-informed precision therapeutics. MDD could be conceptualized as a distributed failure of ionic homeostasis that links neuroinflammation, E/I imbalance, network instability, and impaired adaptive plasticity.

## 1. Introduction

Major depressive disorder (MDD) is among the most prevalent and disabling neuropsychiatric disorders. Although the monoamine hypothesis has generated clinically useful antidepressants and remains important for psychopharmacology, it does not adequately explain the delayed therapeutic onset of conventional agents, the substantial proportion of patients with treatment-resistant depression, or the rapid clinical effects observed after interventions targeting glutamatergic and excitability-related pathways [1,2,3,4]. These limitations provide a broader framework connecting molecular changes with circuit dysfunction and clinical heterogeneity.

Over the past two decades, depression research has increasingly shifted from neurotransmitter deficiency toward synaptic plasticity, neurotrophic signaling, neuroinflammation, and network dysregulation [5,6,7,8]. Ketamine and esketamine have accelerated this conceptual shift by demonstrating that depressive symptoms can be rapidly modified through mechanisms involving glutamate throughput, AMPA receptor signaling, brain-derived neurotrophic factor (BDNF) release, mammalian target of rapamycin (mTOR) activation, synaptogenesis, and reorganization of dysfunctional neural circuits [9,10,11]. These observations support the idea that the depressed brain is not simply depleted of monoamines but is trapped in maladaptive states of excitability, plasticity, and network stability.

Ion channels and transporters are the proximal molecular regulators of excitability. They determine resting membrane potential, action potential threshold, firing frequency, synaptic integration, neurotransmitter release, intracellular calcium signaling, chloride gradients, extracellular potassium buffering, glial inflammatory activation, and oscillatory synchrony. Therefore, channel dysfunction has the potential to translate genetic and environmental risks into altered cellular computations and large-scale network pathologies. This review develops the concept that failure of ionic homeostasis is an upstream and integrative mechanism in MDD.

We define ionic homeostasis failure as a pathological state in which the brain cannot maintain optimal electrochemical gradients or conductance states across neurons and glia. In this state, calcium influx may become maladaptive, potassium conductance and buffering may fail to stabilize firing, chloride gradients may weaken gamma-aminobutyric acid (GABA)-ergic inhibition, and purinergic/glial channels may convert stress signals into inflammatory cascades. The resulting disturbance of ionic equilibrium is proposed to drive excitation–inhibition imbalance, impaired neuroplasticity, and network instability across corticolimbic circuits.

**Figure 1:**
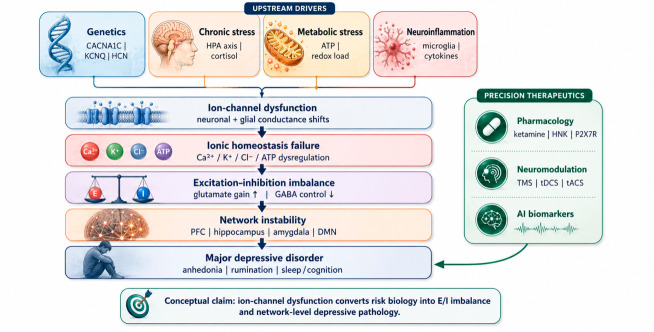
A unified ionic homeostasis framework for major depressive disorder. Upstream genetic, stress, metabolic, and neuroinflammatory drivers converge on neuronal and glial ion-channel dysfunction, causing ionic homeostasis failure, E/I imbalance, network instability, depressive symptoms, and mechanism-guided precision therapeutics.

The review addresses mechanistic and translational aspects. We integrated neuronal channels, glial channels, E/I balance, network oscillations, and therapeutic strategies into a single-system-level framework. We emphasize that the channels with the strongest relevance to MDD and antidepressant pharmacology are CACNA1C/Cav1.2, TREK-1, KCNQ, NKCC1/KCC2, HCN, transient receptor potential (TRP)/acid-sensing ion channels (ASICs), P2X7R, Kir4.1, and AQP4. These mechanisms are connected to ketamine-adjacent pharmacology, precision neuromodulation, and AI/multi-omics-based target discovery. The conceptual architecture is illustrated in Figure 1.

## 2. Ionic Homeostasis and Excitation–Inhibition Balance in Depression

The healthy brain operates within a dynamic range in which excitatory and inhibitory forces are continuously balanced. Glutamatergic projection neurons provide an excitatory drive, whereas GABAergic interneurons, including parvalbumin- and somatostatin-positive subtypes, provide temporal and spatial inhibitory control [12,13]. The E/I balance is not a static ratio but a multiscale property emerging from membrane conductance, synaptic transmission, dendritic integration, glial buffering, and network oscillations.

Ionic homeostasis is a physical substrate for the E/I balance. Sodium, potassium, calcium, chloride, and proton gradients are maintained by pumps, transporters, and channels at high energetic cost [14,15,16]. These gradients define the driving forces of action potentials, synaptic currents, and intracellular signaling. Even modest perturbations can shift neurons away from optimal criticality, thereby altering gain control, synchrony, plasticity thresholds, and susceptibility to maladaptive network states.

Convergent evidence supports altered inhibitory tone, abnormal glutamatergic signaling, and disrupted oscillatory dynamics in MDD. Postmortem and imaging studies have reported GABAergic deficits, altered glutamate/GABA levels, interneuron-related abnormalities, and changes in corticolimbic circuits [17,18,19,20]. EEG and computational studies further suggest that MDD is associated with altered theta/gamma dynamics and deviations from optimal network criticality [21]. These findings are consistent with a model in which depression reflects a distributed failure of network regulation, rather than a uniform increase or decrease in activity.

The topographic E/I imbalance is particularly important. The prefrontal cortex may show reduced cognitive control and impaired flexibility, the hippocampus may show stress-sensitive plasticity deficits, the amygdala may show exaggerated salience and threat responsiveness, and the default mode network may show hyperconnectivity linked to rumination [22,23,24,25]. Thus, the E/I imbalance in MDD should be considered a region- and cell type-specific dysregulation rather than a single global state.

The ion channels provide mechanistic entry points for this topography. Calcium channels couple depolarization to gene expression and structural remodeling; potassium channels stabilize firing and shape adaptation; chloride transporters determine whether GABA is inhibitory; HCN channels influence theta timing and dendritic integration; TRP and ASIC channels link stress, pain, pH, and inflammatory signaling; and glial channels regulate extracellular potassium, water flux, cytokine release, and synapse remodeling [24,25,26]. Together, these systems define the ionic conditions under which the E/I balance is maintained or lost.

**Figure 2:**
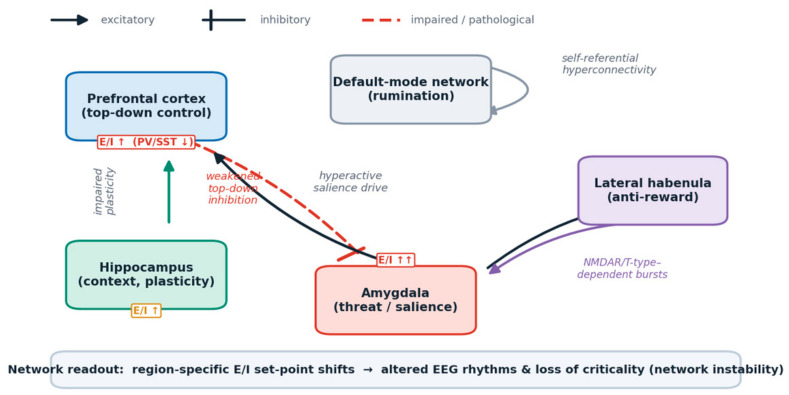
Topographic E/I imbalance across depression-associated networks. E/I dysregulation is represented as region- and network-specific rather than a uniform global state, linking prefrontal control, default-mode rumination, hippocampal plasticity, amygdala salience, lateral habenula anti-reward signaling, EEG/criticality readouts, and set-point shifts. Upward and downward arrows indicate relative increases and decreases, respectively, in the indicated process, activity, or network state. EEG, electroencephalogram.

Therefore, the central claim of this review is hierarchical: upstream genetic, stress, metabolic, and inflammatory factors perturb ion-channel systems; these perturbations produce ionic homeostasis failure, which destabilizes the E/I balance, and E/I imbalance manifests as network dysfunction and depressive symptoms. This model does not replace monoamines, neurotrophic signaling, or inflammation but organizes them within an excitability-centered framework. The region-specific logic of this model is illustrated in Figure 2.

## 3. Calcium Channels and Stress-Related Neuroplasticity

Calcium ions are universal intracellular messengers that couple membrane activity with neurotransmitter release, kinase signaling, transcriptional programs, dendritic remodeling, and survival pathways. Because calcium signaling is powerful and potentially toxic, intracellular calcium is tightly controlled by voltage-gated calcium channels, ligand-gated receptors, intracellular stores, pumps, and exchangers [27,28,29,30].

Dysregulated calcium influx impairs plasticity, enhances excitotoxic stress, and alters stress responsiveness. Calcium signaling is also a convergent vulnerability in psychiatric diseases, in which adaptive activity-dependent transcription can be converted into maladaptive stress signaling when homeostatic control fails [31].

Voltage-gated calcium channels, particularly L-type Cav1.2 channels encoded by CACNA1C, have attracted substantial interest in psychiatric genetics. CACNA1C variants have been associated across diagnostic boundaries, including bipolar disorder, schizophrenia, and depression-related phenotypes [32,33,34,35]. This cross-disorder pattern is consistent with the concept that calcium channel dysregulation affects fundamental dimensions of brain function, including excitability, plasticity, and stress adaptation, rather than a single categorical diagnosis.

Functional studies suggest that psychiatric risk variants within CACNA1C can alter calcium channel activity, intracellular calcium dynamics, and downstream transcriptional signaling [35,36,37,38]. In human-induced neuronal systems and animal models, altered Cav1.2 function can influence dendritic development, activity-dependent gene expression, fear learning, stress susceptibility, and synaptic function [39,40]. These observations place CACNA1C at the top of the molecular hierarchy, linking genetic risk to circuit-level maladaptation.

Calcium signaling intersects with the BDNF, CREB, CaMK, and mTOR pathways [30,31]. In adaptive states, calcium-dependent transcription supports learning and synaptic remodeling. However, under chronic stress, glucocorticoid exposure and metabolic load may convert calcium signaling into maladaptive dendritic atrophy, synapse loss, and impaired resilience in the prefrontal cortex and hippocampus [4]. Therefore, ions that support plasticity can cause damage when homeostatic regulation fails.

**Figure 3:**
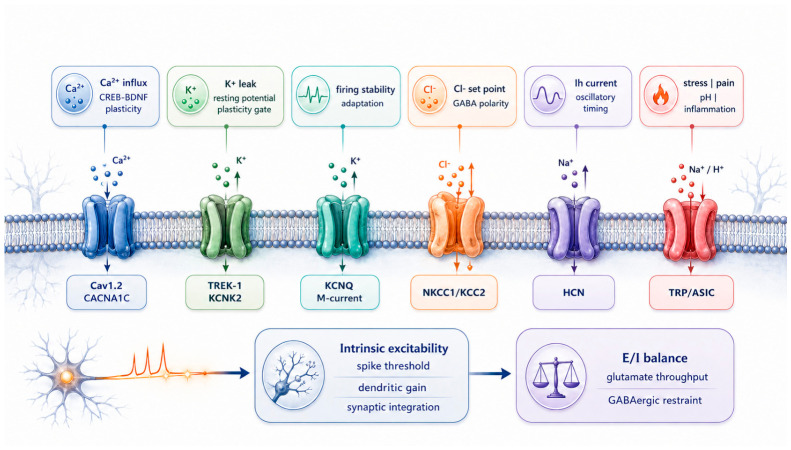
Neuronal ion channels governing excitability and E/I balance. Voltage-gated calcium channels, potassium channels, chloride regulators, HCN channels, and TRP/ASIC channels converge on intrinsic excitability, plasticity thresholds, inhibitory tone, oscillatory timing, and network-level E/I balance. HCN, hyperpolarization-activated cyclic nucleotide-gated; TRP, transient receptor potential; ASIC, acid-sensing ion channel.

Therapeutic exploitation of calcium channels remains challenging because Cav1.2 channels are essential in the cardiovascular system. Therefore, a direct systemic blockade may be unsuitable for treating depression unless brain selectivity, circuit targeting, or pathway-specific modulation can be achieved. Nevertheless, CACNA1C illustrates the central theme of this review: psychiatric risk can act through the ion channel regulation of cellular excitability, converting molecular variation into network vulnerability. The major neuronal channel systems supporting this framework are summarized in Figure 3 and Table 1.

## 4. Potassium Channels and Neuronal Stability

Potassium channels are fundamental stabilizers of neuronal activity. By shaping resting membrane potential, afterhyperpolarization, spike-frequency adaptation, burst firing, dendritic integration, and extracellular potassium dynamics, they set the excitability range of neurons and glial networks [41,42,43,44]. In the ionic homeostasis framework, potassium channels counterbalance the depolarizing sodium and calcium currents and protect networks from runaway excitation or maladaptive hyporesponsiveness.

TREK-1, encoded by KCNK2, is a two-pore-domain potassium channel that provides background leak conductance and responds to membrane stretch, temperature, pH, lipids, and pharmacological agents [45,46,47]. Genetic deletion or pharmacological inhibition of TREK-1 produces antidepressant-like phenotypes in preclinical models [41]. Therefore, TREK-1 has become a key example of how modulation of background potassium conductance can influence mood-related behavior without acting primarily through monoamine reuptake.

KCNQ channels generate M currents that suppress repetitive firing and stabilize the membrane potential. Although best known in epilepsy and excitability disorders, KCNQ channels are relevant to affective circuits because they regulate pyramidal neuron gain, interneuron excitability, and oscillatory stability [48,49,50,51]. Abnormal KCNQ function could plausibly contribute to either excessive limbic responsiveness or impaired cortical flexibility, depending on the circuit location and cell type.

Kir channels, especially astrocytic Kir4.1, extend potassium channel biology beyond neurons. Astrocytic Kir4.1, which mediates spatial potassium buffering, helps maintain extracellular potassium within a range compatible with stable network functions [44,52]. If Kir4.1-mediated buffering fails, extracellular potassium can accumulate, depolarize neurons, increase network excitability, and amplify E/I imbalance [53,54,55,56]. This mechanism directly links glial dysfunction to circuit instability. In addition, calcium-activated potassium channels (BK and SK) shape the afterhyperpolarization and spike-frequency adaptation that govern repetitive firing, further tuning neuronal excitability relevant to network stability [57,58].

Thus, the potassium channel links these two therapeutic concepts. First, neuronal potassium channels such as TREK-1 and KCNQ can tune intrinsic excitability and plasticity thresholds. Second, glial potassium channels, such as Kir4.1, maintain the extracellular ionic conditions required for stable computations. Depression may involve both altered neuronal responsiveness and impaired glial buffering, making K^+^ homeostasis the central pillar of the proposed framework.

## 5. Chloride Homeostasis and GABAergic Dysfunction

GABAergic inhibition depends on the chloride gradient. The polarity and strength of GABA_A receptor-mediated currents are determined by intracellular chloride concentration, which is regulated mainly by NKCC1-mediated chloride import and KCC2-mediated chloride extrusion [54,59,60,61]. Axonal and somatodendritic NKCC1 can further shape local chloride microdomains and inhibit their efficacy in principal cortical neurons [62]. In mature neurons, high KCC2 activity maintains low intracellular chloride levels, allowing GABA to hyperpolarize or shunt postsynaptic membranes.

If the NKCC1/KCC2 balance shifts toward chloride accumulation, GABAergic transmission becomes less inhibitory and may even become depolarized under certain conditions [60,61,63,64]. This process is well established in neurodevelopment and epilepsy but is increasingly relevant to psychiatric disorders, where subtle weakening of inhibitory tone can alter network gain and emotional regulation. Therefore, chloride homeostasis provides a direct biophysical pathway from transporter dysfunction to an E/I imbalance.

Chloride homeostasis is thermodynamically coupled to potassium homeostasis [44,52]. Since KCC2 extrudes Cl^−^ using an outwardly directed K^+^ gradient, extracellular K^+^ accumulation caused by impaired astrocytic Kir4.1-mediated buffering can reduce the driving force for KCC2-dependent Cl^−^ extrusion [54,60,61]. As intracellular Cl^−^ increases, GABAA receptor-mediated signaling becomes less hyperpolarized and may become functionally depolarized in vulnerable circuits [55,56]. This provides a direct biophysical bridge between astrocytic potassium-buffering failure and weakened synaptic inhibition, positioning glial dysfunction upstream of the E/I imbalance [65].

The evidence of GABAergic dysfunction in depression includes reduced cortical GABA concentrations, altered expression of GABA-related genes, interneuron abnormalities, and altered inhibitory signaling in corticolimbic regions [17,18,19,20]. Chloride dysregulation offers a mechanistic explanation for the failure of GABAergic synapses even when GABA is present [66]. The problem may not only be neurotransmitter availability but also the electrochemical gradient that determines the inhibitory efficacy.

Therapeutically, chloride restoration remains underdeveloped in depression compared to that in epilepsy and neurodevelopmental disorders. However, the mechanistic rationale is strong: re-establishing KCC2-dominant chloride extrusion or reducing excessive NKCC1 activity could restore the inhibitory tone and stabilize network function. This strategy should be considered cautiously because chloride gradients are cell type-, age-, and region-dependent. However, it represents a key example of ionic homeostasis repair.

## 6. Hyperpolarization-Activated Cyclic Nucleotide-Gated (HCN) Channels, Oscillatory Activity, and EEG Biomarkers

HCN channels generate Ih currents that influence resting membrane potential, dendritic integration, pace-making, and resonance [67,68,69,70]. HCN channels are particularly important for rhythmic activity and temporal coordination, making them relevant to EEG abnormalities and network criticality in depression.

Depression is associated with altered oscillatory signatures across theta, alpha, beta, and gamma bands [71,72,73,74]. Although these signatures are not specific to a single-channel family, HCN channels are positioned to influence theta-gamma coupling, dendritic filtering, and network timing. Therefore, dysregulated HCN function could contribute to abnormal synchronization in the prefrontal, hippocampal, and default-mode circuits.

EEG-derived biomarkers may provide functional readouts of ion-channel-linked excitability. A patient with reduced inhibitory control, altered theta/gamma balance, or abnormal criticality may not require the same treatment as a patient with an inflammatory or glial-dominant pathology. Therefore, HCN channels serve as a conceptual bridge between molecular excitability and non-invasive biomarkers.

## 7. TRP and ASIC Channels in Stress, Pain, Inflammation, and Emotional Behavior

The transient receptor potential (TRP) and acid-sensing ion channels (ASICs) expand the ionic homeostasis framework beyond classical synaptic excitability. These channels detect temperature, pH, osmotic stress, lipid mediators, inflammatory signals, and tissue injury [73,74,75,76]. Because depression is frequently comorbid with pain, anxiety, inflammation, and autonomic dysregulation, sensory stress channels may contribute to affective symptoms in specific patient subsets.

TRPV1 is a notable example of such a phenomenon. It is expressed in sensory neurons and central regions and participates in pain, stress responsivity, and emotional behavior [73,74,75]. TRPV1 activation influences glutamatergic transmission, neuropeptide release, and inflammatory signaling. Although the literature on depression is less mature than that on CACNA1C or TREK-1, TRP channels may be relevant for patients in whom pain, inflammation, and mood symptoms are tightly coupled.

ASIC1a responds to extracellular acidification and is involved in fear, anxiety, synaptic plasticity, and stress-sensitive behavior [76,77,78,79]. ASIC1a deletion can also alter behavioral responses to stress and drugs, supporting the broader role of pH-sensitive signaling in affective regulation [80]. Changes in local pH can occur during intense activity, inflammation, ischemia, or metabolic stress, thereby linking extracellular homeostasis to emotional behaviors. Therefore, ASIC and TRP channels should be treated as modulators of stress-coupled excitability rather than as core depression-specific channels.

The strongest evidence in this review comes from calcium, potassium, chloride, and glial channels. The TRP and ASIC channels were included because they broaden the framework to include pain, pH, inflammation, and interoceptive stress, all of which are clinically relevant to MDD heterogeneity. This broader stress-sensing context also links sensory ion channels to purinergic and inflammatory signaling [81,82].

## 8. Glial Ion Channels and Neuroinflammation

The most distinctive feature of the ionic homeostasis framework is the presence of glial ion channels. Astrocytes and microglia are not passive support cells; they regulate extracellular potassium, glutamate clearance, water flux, inflammatory signaling, synapse remodeling, neurovascular coupling, and glymphatic clearance [83,84,85,86]. Because these processes shape the ionic environment in which neurons operate, glial dysfunction can precede and amplify the neuronal E/I imbalance.

Microglial P2X7 receptors provide a direct mechanistic link between extracellular danger signals and inflammatory ion flux. Chronic stress, tissue damage, metabolic strain, and impaired clearance can increase extracellular ATP [86,87,88,89]. Sustained ATP stimulation activates P2X7R, promotes cation flux and potassium efflux, facilitates NLRP3 inflammasome assembly, and increases maturation and release of IL-1β and other inflammatory mediators [90,91,92,93]. These cytokines alter synaptic plasticity, glutamate handling, neurotrophic signaling, and inhibitory control.

The P2X7R–NLRP3 axis is especially valuable for this review because it unites ion channel biology and neuroinflammation. P2X7R is not merely an inflammatory receptor; it is also an ion channel whose activation changes ionic gradients [83,84,85]. Thus, inflammatory activation is coupled with failure of ionic homeostasis at the membrane conductance level. Thus, P2X7R is a central mechanistic node linking stress, glial activation, cytokine release, and network dysfunction.

Astrocytic Kir4.1 is another critical node. Under physiological conditions, astrocytes buffer extracellular potassium released during neuronal firing, preventing excessive depolarization and maintaining stable network excitability [94,95,96,97]. Reduced Kir4.1 function can impair potassium clearance, elevate extracellular potassium, and promote hyperexcitability [55,56,65]. In affective circuits, such a glial buffering failure can shift the E/I balance toward unstable states and reduce the precision of neural coding.

AQP4 contributes to water homeostasis and glymphatic function. Perivascular AQP4 polarization supports cerebrospinal–interstitial fluid exchange and clearance of metabolites [98,99,100,101]. Sleep disruption, inflammation, vascular dysfunction, and astrocytic pathology may impair AQP4 organization and glymphatic function. Since sleep disturbances and inflammatory burdens are common in depression, AQP4-mediated clearance may be relevant to chronicity and treatment resistance.

Impaired AQP4-dependent glymphatic clearance may be particularly relevant in MDD, where sleep disruption is common [99,100,101,102]. Because slow-wave sleep facilitates interstitial solute removal, persistent sleep fragmentation can increase the metabolic burden and local acidification within the corticolimbic circuits. Such changes may engage pH-sensitive channels, including ASIC1a, thereby increasing excitability in the amygdala-linked fear and negative-affect circuits [77,78]. Although the AQP4–glymphatic–ASIC axis remains hypothetical in depression, it offers a testable framework linking sleep disturbances, metabolic stress, interstitial homeostasis, and affective circuit instability.

P2X7R, Kir4.1, and AQP4 define the glial triad through inflammatory gating, potassium buffering, and water/clearance regulation. Failure of this triad can create a self-reinforcing loop in which ATP release activates microglia, cytokines impair synapses, astrocytes fail to buffer extracellular potassium, glymphatic clearance decreases, and neuronal networks become unstable. This glia-driven failure of ionic homeostasis may help explain why stress-related depression becomes persistent and why purely monoaminergic therapies are often insufficient.

**Figure 4:**
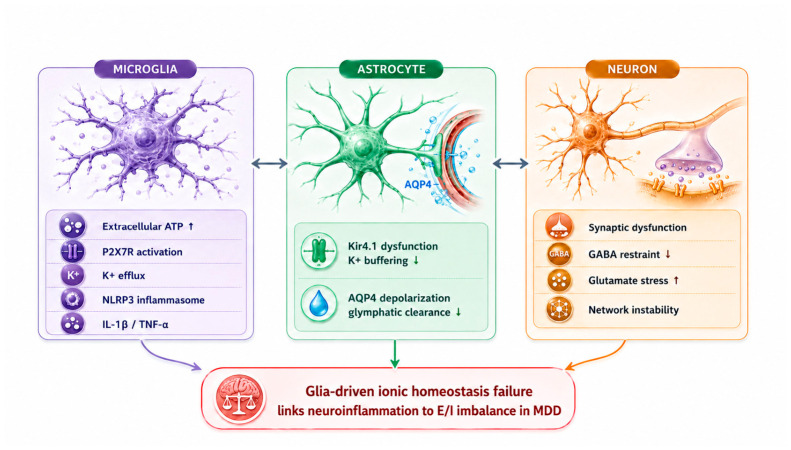
Microglia–astrocyte–neuron crosstalk in ionic homeostasis failure. P2X7R–NLRP3 signaling, Kir4.1-dependent potassium buffering, AQP4-mediated water/clearance regulation, and synaptic dysfunction are integrated into a self-reinforcing glia-driven pathological loop. Upward arrows indicate increased activation or levels, whereas downward arrows indicate reduced function, buffering, or clearance.

Glial channels offer targets that differ from those of conventional neuronal receptors. P2X7R antagonists, NLRP3 inhibitors, strategies to restore astrocytic potassium buffering, and approaches that improve AQP4 polarization or glymphatic function may complement antidepressants that affect neuronal plasticity. The key translational challenge is selectivity. Glial pathways are widely involved in immune defense and homeostasis; therefore, future therapies will require brain-, cell-, and state-dependent precision. The glia-driven pathological loop is shown in Figure 4.

## 9. Ion-Channel-Targeted Therapeutics

If MDD reflects ionic homeostasis failure, therapeutic success should be defined not only by symptom improvement but also by the restoration of adaptive excitability, E/I balance, plasticity, and network stability [103,104,105,106]. This perspective reframes the discovery of antidepressant drugs based on monoamine enhancement of excitability repair.

Ketamine provided the basis for this paradigm. Although commonly described as an NMDA antagonist, its antidepressant effects involve downstream glutamate throughput, AMPA activation, BDNF release, mTOR signaling, synaptogenesis, and network reconfiguration [103,104,105,106]. Synaptic and rapid-acting antidepressant models further support the view that ketamine reopens adaptive plasticity states [107,108]. Clinical studies on ketamine and esketamine have demonstrated rapid improvements in treatment-resistant depression [109,110,111,112]. Meta-analytical evidence also supports acute reduction in suicidal ideation after ketamine administration, strengthening the translational relevance of excitability- and plasticity-based antidepressant mechanisms [113]. Ketamine demonstrates that rapid clinical improvement can occur when a pathological network state is destabilized and the plasticity window is opened. Notably, the precise contribution of NMDA receptor antagonism itself remains debated: whereas NMDA receptor-dependent burst firing in the lateral habenula has been proposed as a direct driver of ketamine’s rapid antidepressant action [11,114], other evidence emphasizes NMDA receptor-independent, metabolite- and AMPA receptor-driven contributions, indicating that these rapid effects likely arise from convergent rather than singular mechanisms.

Hydroxynorketamine (HNK), particularly (2R,6R)-HNK, has generated interest because it may retain antidepressant-like efficacy while reducing the dissociative and psychotomimetic liabilities associated with ketamine [105,106]. TREK-1 blockade is especially relevant to this review because it links ketamine-adjacent pharmacology to background potassium conductance and intrinsic excitability [41,45]. This connection should be treated as promising but requires careful verification and replication. In particular, whether (2R,6R)-HNK attains brain concentrations sufficient to engage TREK-1 at behaviorally active doses and whether TREK-1 blockade is necessary for its antidepressant-like effects remain unresolved and warrant cautious interpretation [115,116].

TREK-1 itself is a compelling target because its blockade can increase neuronal responsiveness and facilitate plasticity. However, therapeutic modulation is required to avoid nonspecific hyperexcitability. The ideal antidepressant strategy may not be maximal channel inhibition, but state-dependent normalization of conductance within specific circuits.

P2X7R antagonism is an anti-inflammatory ion channel strategy. By limiting ATP-driven inflammasome activation, P2X7R inhibition may reduce IL-1β release, dampen microglial activation, improve synaptic function, and restore network stability [83,84,85,86]. Such interventions may be particularly useful for the treatment of inflammatory depression or in patients with elevated immune biomarker levels. However, this promise must be tempered by the so-far mixed clinical track record of P2X7R antagonists in inflammatory and neuropsychiatric conditions, which underscores the need to confirm central target engagement and to stratify patients by inflammatory status [117,118].

Chloride-restoration strategies offer alternative routes. Enhancing KCC2 function or reducing pathological NKCC1-driven chloride accumulation could strengthen the inhibitory tone and reduce the E/I imbalance. Although the clinical translation to depression remains early, the mechanistic logic is strong and aligns with the broader goal of ionic gradient restoration.

**Figure 5:**
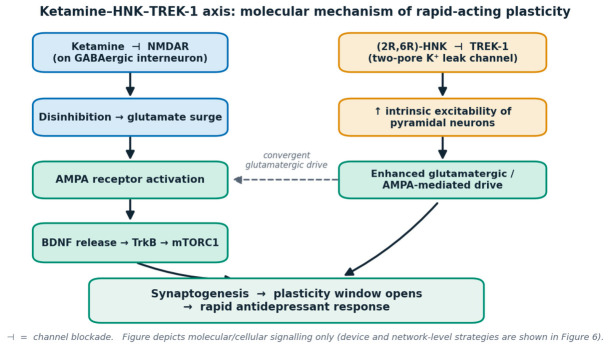
Ketamine–HNK–TREK-1 axis and plasticity-window therapeutics. Rapid-acting pharmacology is organized from NMDA/AMPA-BDNF-mTOR signaling through the HNK/TREK-1-linked excitability branch, converging on synaptogenesis and the opening of the plasticity window. The symbol “⊣” indicates channel blockade. This figure depicts molecular and cellular signaling; device- and network-level strategies are shown in Figure 6.

**Figure 6:**
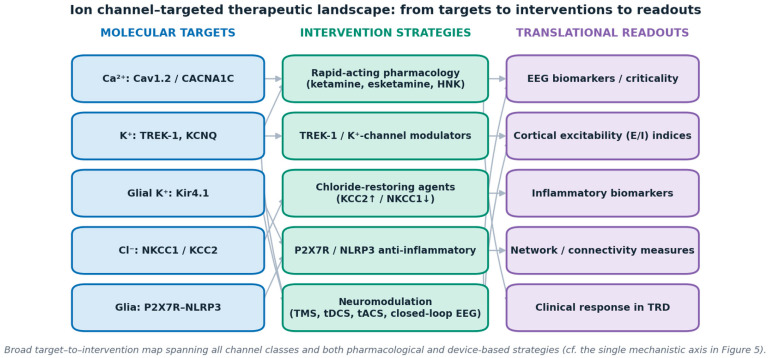
Ion-channel-targeted therapeutic landscape in depression. Disease modules are connected to target classes, intervention strategies, and translational readouts relevant to mechanism-guided antidepressant development. Upward and downward arrows indicate increased and decreased target activity or function, respectively. This broad target-to-intervention map spans multiple channel classes and both pharmacological and device-based therapeutic strategies; the single mechanistic ketamine–HNK–TREK-1 axis is shown separately in Figure 5.

Therefore, next-generation antidepressants may combine molecular precision and network guidance. Pharmacological interventions can open or stabilize plasticity windows, whereas TMS, tDCS, tACS, or closed-loop EEG-guided stimulation direct plasticity toward healthy network states. This drug–device combination model may reduce the required drug doses, limit systemic adverse effects, and improve circuit specificity. The ketamine–HNK–TREK-1 mechanistic hierarchy is summarized in Figure 5. The broader target-to-intervention landscape is summarized in Figure 6 and Table 2.

## 10. AI and Multiomics-Based Ion-Channel Target Discovery

Depression is a heterogeneous condition. Single-channel and single-drug models are not suitable for all patients. Multiomics technologies provide tools to map this heterogeneity across genetic risks, transcriptional regulation, cell type specificity, spatial localization, proteomic state, metabolite burden, and digital/physiological biomarkers [119,120,121,122].

A GWAS can identify risk loci enriched in neuronal and synaptic genes, including ion-channel-associated pathways [119,120,121,122]. Single-cell RNA sequencing can be used to determine the neuronal or glial populations that express disease-relevant channel subunits [123,124]. Spatial transcriptomics and computational cell-type annotation can place these changes within corticolimbic microcircuits and disease-relevant cellular populations [125,126]. Proteomics and metabolomics can reveal whether changes in channel transcripts translate into altered protein abundance, inflammatory signaling, energy stress, or ionic–metabolic coupling.

Artificial intelligence can integrate these data layers to generate patient-specific ion channel signatures. For example, one patient may show a calcium/plasticity-dominant signature, glial inflammatory signature, chloride/GABAergic signature, and oscillatory HCN-related signature. These signatures can be used to inform drug selection, neuromodulation, and biomarker monitoring.

**Figure 7:**
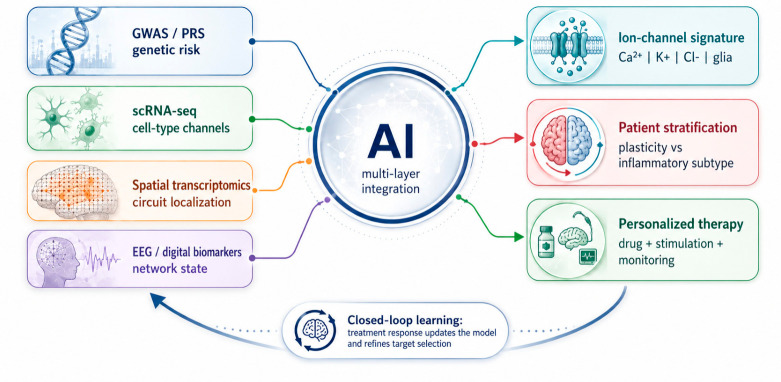
AI-enabled precision psychiatry for ion-channel target discovery. Genomics, single-cell and spatial transcriptomics, EEG/digital biomarkers, and machine learning are integrated to identify patient-specific ion-channel signatures and adaptive treatment strategies. EEG, electroencephalogram.

The practical goal of precision psychiatry is to stratify patients by mechanism rather than by symptom score alone. In such a system, EEG and digital biomarkers provide real-time network-state readouts, multiomics identify molecular vulnerabilities, and AI models recommend interventions that restore ionic homeostasis with minimal off-target burden [127,128]. The genetic and multiomics evidence nodes are summarized in Table 3, and the AI-enabled precision framework is summarized in Figure 7 and Table 4.

## 11. Future Perspectives

Several issues must be resolved before the ionic homeostasis framework can be used in clinical programs. First, the causality must be tested. Many ion channel changes observed in depression may reflect compensation rather than a primary pathology. Longitudinal, cell type-specific, and circuit-resolved studies are needed to determine when channel dysfunction initiates disease and when it emerges as an adaptation.

Second, neuronal and glial mechanisms must be integrated experimentally. Neurons, astrocytes, and microglia are often studied separately; however, ionic homeostasis is fundamentally multicellular. Future studies should combine electrophysiology, imaging, single-cell transcriptomics, spatial profiling, and behavioral assays to map how glial buffering and inflammatory activation alter the neuronal E/I balance.

Third, the pharmacological specificity remains a major challenge. Ion channels are widely expressed in the brain and periphery, and many are essential for cardiovascular, immune, and sensory functions. Successful therapies require subtype selectivity, brain targeting, allosteric modulation, state-dependent pharmacology, or drug–device combinations that reduce systemic exposure.

Fourth, non-canonical ion channels should be carefully explored. Calcium-activated chloride channels such as ANO1/TMEM16A, other glial transport systems, and mechanosensitive channels may participate in the neuroinflammatory or interoceptive dimensions of mood disorders. These candidates should be framed as future opportunities rather than established antidepressant targets.

Closed-loop precision neuromodulation may be a key component of ion-channel-targeted therapeutics. Pharmacological intervention can open or stabilize the plasticity window; however, EEG-guided stimulation may help direct the trajectory of network reorganization. Patients with prefrontal inhibitory control deficits, theta-band instability, or abnormal gamma synchronization are unlikely to require identical stimulation logic. In principle, real-time network-state biomarkers can be used to adapt to the stimulation frequency, phase, and circuit targets in a patient-specific manner. However, these strategies should be considered experimental and require prospective biomarker-guided clinical validation.

## 12. Conclusions

The evidence reviewed here supports a broader interpretation of MDD as a systemic disorder of ionic homeostasis. Monoaminergic abnormalities remain clinically relevant but do not fully account for treatment resistance, rapid antidepressant mechanisms, neuroinflammatory subtypes, glial pathology, or network instability.

Ion channels and transporters provide a mechanistic bridge between molecular risks and circuit dysfunction. CACNA1C/Cav1.2 links genetic susceptibility to calcium-dependent plasticity and stress signaling; TREK-1 and KCNQ regulate potassium conductance and neuronal stability; NKCC1/KCC2 determine inhibitory chloride gradients; HCN channels shape network timing; TRP and ASIC channels link pain, pH, and stress; and glial P2X7R, Kir4.1, and AQP4 regulate inflammatory gating, potassium buffering, and clearance.

We propose that neuronal and glial ion channel dysfunction converges upon the failure of ionic homeostasis, producing E/I imbalance, neuroinflammation, network instability, and impaired neuroplasticity. This framework provides a unified model for the discovery and precision of next-generation antidepressants in psychiatry. Future therapeutic strategies should aim not only to increase monoamines but also to restore the ionic and network conditions that permit adaptive mood regulation.

## Figures and Tables

**Table 1:** Major ion channels implicated in depression.

Channel/Transporter	Cell Type	Primary Function	Depression Relevance
CACNA1C/Cav1.2	Pyramidal neurons, interneurons	Ca^2+^ influx; activity-dependent transcription; plasticity	Genetic risk; stress sensitivity; plasticity deficits
TREK-1/KCNK2	Neurons; some glia	Background K^+^ conductance; membrane stability	Antidepressant-like effects after blockade; HNK-related interest
KCNQ	Neurons	M-current; suppression of repetitive firing	Network stability; excitability modulation
Kir4.1	Astrocytes	Extracellular K^+^ buffering	Glial contribution to hyperexcitability
NKCC1/KCC2	Neurons	Intracellular Cl^−^ set-point; GABA polarity	Direct mechanism for impaired inhibition
HCN	Neurons	Ih current; dendritic integration; oscillatory timing	EEG rhythm and criticality linkage
TRPV1/TRPC	Sensory and central neurons; glia	Stress, pain, inflammatory signaling	Mood–pain–inflammation interface
ASIC1a	Neurons	Extracellular acid sensing	Fear, stress, emotional behavior
P2X7R	Microglia, immune cells	ATP-gated cation channel; inflammasome activation	Neuroinflammatory depression subtype
AQP4	Astrocytic endfeet	Water flux; glymphatic clearance	Sleep/inflammation/clearance axis

**Table 2:** Current and emerging therapeutic strategies.

Agent/Approach	Target	Mechanism	Development Stage
Ketamine/esketamine	NMDA receptor modulation; downstream AMPA-BDNF-mTOR	Rapid plasticity and network reconfiguration	Clinical use for TRD/suicidality under strict monitoring
(2R,6R)-HNK	Ketamine metabolite; proposed TREK-1/excitability-linked effects	Potential plasticity without full ketamine liabilities	Preclinical/early translational; mechanism evolving
TREK-1 blockers	KCNK2/TREK-1	Increase neuronal responsiveness and plasticity	Preclinical target
P2X7R antagonists	P2RX7	Reduce ATP-driven inflammasome activation	Potential inflammatory-depression strategy
NKCC1/KCC2 modulators	Chloride transporters	Restore GABAergic inhibition	Experimental/repurposing concept
tACS/tDCS/TMS	Network excitability, oscillations	Bias plasticity toward healthier circuit states	Clinical neuromodulation; combination opportunity

**Table 3:** Genetic and multiomics evidence nodes.

Gene/Target	Evidence Type	Interpretation
CACNA1C	GWAS, iPSC neurons, animal models	Cross-disorder psychiatric risk; Cav1.2 function; stress/plasticity
KCNK2/TREK-1	Knockout and pharmacological blockade	Antidepressant-like behavioral effects; K^+^ leak modulation
KCNQ family	Electrophysiology, excitability disorders, pharmacology	M-current regulation; possible circuit-stabilizing target
SLC12A2/SLC12A5	Transporter biology; chloride-gradient models	NKCC1/KCC2 balance determines inhibitory efficacy
P2RX7	Inflammation genetics; microglial models	ATP-P2X7R-NLRP3-IL-1β cascade
KCNJ10/Kir4.1	Astrocyte physiology; glial pathology	K^+^ buffering and network stabilization
AQP4	Astrocyte/glymphatic studies	Water clearance, sleep, and neuroinflammatory burden

**Table 4:** Precision psychiatry platforms.

Platform	Primary Application	Use in Ionic-Homeostasis Framework
GWAS/polygenic scoring	Identify inherited risk and channel-enriched loci	Mechanism-informed stratification
Single-cell RNA-seq	Map channel expression by cell type	Neuron/glia-specific target selection
Spatial transcriptomics	Localize molecular dysfunction in circuits	Topographic E/I and glial-state mapping
Proteomics/metabolomics	Detect protein abundance and metabolic stress	Functional validation of transcriptomic candidates
EEG/MEG	Measure oscillations and criticality	Closed-loop stimulation and treatment monitoring
AI integration	Fuse multiomics, imaging, EEG, and symptoms	Patient-specific ion-channel signatures

## Data Availability

No new data were created or analyzed in this study. Data sharing is not applicable to this article.

## Abbreviations

The following abbreviations are used in this manuscript:

AQP4	aquaporin-4
ASIC	acid-sensing ion channel
BDNF	brain-derived neurotrophic factor
BK	large-conductance calcium-activated potassium channel
CACNA1C	calcium voltage-gated channel subunit alpha1 C
Cav	voltage-gated calcium channel
Cl^−^	chloride
DMN	default mode network
EEG	electroencephalography
E/I	excitation–inhibition
GABA	gamma-aminobutyric acid
HCN	hyperpolarization-activated cyclic nucleotide-gated channel
HNK	hydroxynorketamine
IJMS	International Journal of Molecular Sciences
KCC2	potassium–chloride cotransporter 2
Kir	inwardly rectifying potassium channel
MDD	major depressive disorder
mTOR	mammalian target of rapamycin
NKCC1	sodium–potassium–chloride cotransporter 1
NLRP3	NLR family pyrin domain-containing 3
P2X7R	purinergic P2X7 receptor
PFC	prefrontal cortex
SK	small-conductance calcium-activated potassium channel
TMS	transcranial magnetic stimulation
tACS	transcranial alternating-current stimulation
tDCS	transcranial direct-current stimulation
TRD	treatment-resistant depression
TREK-1	TWIK-related potassium channel 1
TRP	transient receptor potential
